# Case Report: Two cases of mammary intraductal papillary adenomas in nulliparous aged dairy cattle

**DOI:** 10.3389/fvets.2026.1766623

**Published:** 2026-03-11

**Authors:** Olanrewaju Ifeoluwa Fatola, Anne Balkema-Buschmann, Martin H. Groschup, Reiner Ulrich

**Affiliations:** 1 Institute of Novel and Emerging Infectious Diseases, Friedrich-Loeffler-Institut, Greifswald - Insel Riems, Germany; 2 Department of Veterinary Anatomy, Faculty of Veterinary Medicine, University of Ibadan, Ibadan, Nigeria; 3 Department of Experimental Animal Facilities and Biorisk Management, Friedrich-Loeffler-Institut, Greifswald - Insel Riems, Germany; 4 Institute of Veterinary Pathology, Faculty of Veterinary Medicine, Leipzig University, Leipzig, Germany

**Keywords:** cattle, histopathology, immunohistochemistry, mammary gland, neoplasia

## Abstract

Despite the mammary gland’s extended volume, intense metabolic turnover and economic importance in dairy cattle, it is a mystery why mammary neoplasms are so rare in this species. This report describes the gross, histopathological, and immunohistochemical features of two cases of mammary intraductal papillary adenomas in two, nine- and 10-year-old, nulliparous Holstein–Friesian cows that were part of a long-term experiment concerning bovine spongiform encephalopathy (BSE). Necropsy revealed multiple intraductal papillary masses and ductal accumulation of serous fluid and / or suppurative exudate, confined to one mammary quarter in each cow. Microscopically, lesions consisted of well-differentiated papillary proliferations lined by cytokeratin-positive epithelial cells supported by smooth muscle actin–positive myoepithelium and vimentin-positive fibrovascular stroma. A periodic acid–Schiff reaction confirmed an intact basal membrane. No necrosis, invasive growth or metastasis were detected. This case report of two benign mammary neoplasms in aged, nulliparous dairy cows provides empirical evidence for the hypothesis of parity- and lactation-related protection from neoplasia in cattle.

## 1 Introduction

Primary mammary neoplasms in cattle are extremely rare, with only sporadic reports describing benign lesions, such as fibroadenomas, adenomas, and papillomas (1, 2), as well as malignant lesions, such as carcinomas (3–6) and sarcomas (7, 8). Hughes (9) emphasized that the mammary glands of ruminants, although morphologically comparable to the human breast (1, 10), seldom undergo neoplastic transformation. This contrasts sharply with the high prevalence of mammary neoplasms in dogs, cats (11–13), and humans (14, 15), where hormonal and genetic factors play recognized roles in oncogenesis. The low frequency of bovine mammary neoplasms has been attributed to short reproductive lifespans, frequent lactation, and reduced cumulative estrogen exposure (4, 7, 9). Barash (16) proposed that cows demonstrate attenuated activation of key oncogenic pathways compared with carnivores and omnivores. However, the biological basis of this apparent resistance remains incompletely understood.

Recent comparative oncology studies have highlighted significant interspecies differences in susceptibility to mammary neoplasia. Herbivores, including ruminants such as cattle and sheep, as well as non-ruminant herbivores such as horses, exhibit strikingly low rates of mammary neoplasia (16–18). Mammary neoplasms in mares are rare even though many females remain nulliparous throughout life, and mammary neoplasms in small ruminants are infrequently reported, despite the presence of aged, non-breeding animals in non-production settings. These observations suggest that neoplasia-resistance in herbivores extends beyond reproductive history and may reflect shared metabolic, endocrine, and mammary-stem-cell regulatory mechanisms (16).

Today, data on bovine mammary neoplasms remain scarce (2, 19, 20). Most reported cases lack immunohistochemical confirmation or precise morphologic classification. Here, we describe two spontaneous cases of mammary intraductal papillary adenomas in aged, nulliparous Holstein-Friesian cows. The report integrates gross, histopathological, and immunophenotypic features and discusses their diagnostic and comparative relevance within the framework of veterinary and human oncology.

## 2 Case description

### 2.1 Clinical history

Two nulliparous Holstein–Friesian cows, aged nine and 10 years, that were involved in a long-term bovine spongiform encephalopathy (BSE) pathogenesis study (21) at the Friedrich-Loeffler-Institut, Greifswald–Insel Riems, Germany, were examined post-mortem. The experiment was conducted under biosafety level 3** conditions and approved by the State Office for Agriculture, Food Safety and Fisheries, Mecklenburg-Vorpommern, Germany (approval no. 7221.3–1.1-019/10). Both animals were transfused with blood from cattle in the clinical end stage of BSE at the age of 4–6 month and clinically monitored thereafter, according to the study protocol. Case 1: The nine-year-old cow developed mastitis approximately 8 weeks before necropsy, presenting with purulent discharge from the affected quarter. Despite multiple applications of Meloxicam, Amoxicillin, Enrofloxacin, and intramammary Ampicillin–Cloxacillin therapy, clinical signs persisted. Six weeks later, the cow showed systemic illness with fever (40.5 °C) and reduced appetite, and was euthanized following lack of therapeutic response. Case 2: The 10-year-old cow showed no clinical signs before euthanasia at the designated endpoint of the study, but was found at necropsy to have a swollen udder.

### 2.2 Gross findings

**Figure 1:**
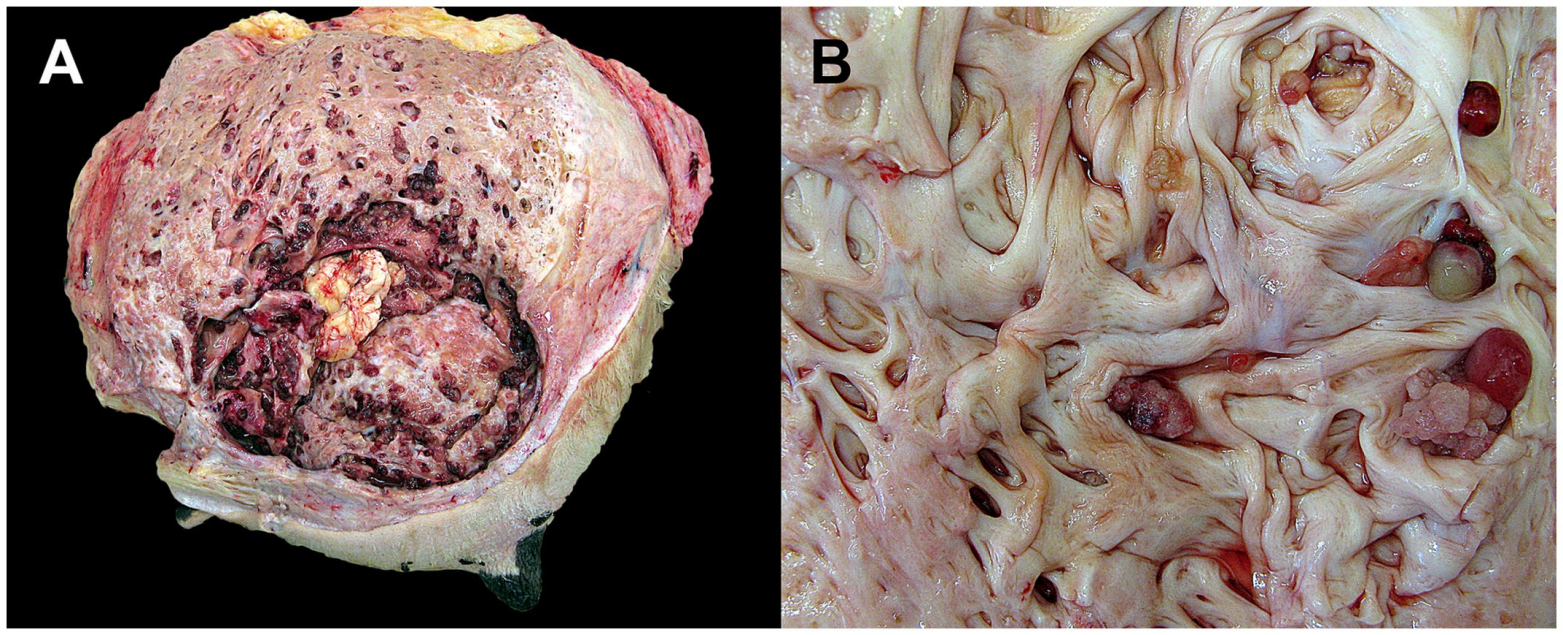
Gross appearance of two cases of multiple intraductal papillary adenomas of the bovine udder. **(A)** Case 1: Marked expansion of the left cranial quarter of the mammary gland due to multiple intraductal and intrasinusoidal papillomatous masses with intraluminal accumulation of serous fluid containing multiple caseous clots. **(B)** Case 2: Moderate expansion of the right cranial quarter of the mammary gland caused by multiple intraductal papillomatous and cystic masses with intraluminal accumulation of serous fluid.

In case 1, the left cranial quarter of the udder was markedly enlarged. The lumen of the lactiferous sinus was cystically dilated and contained multiple, cauliflower-like, intraluminal papillomatous masses, intraluminal accumulation of serous fluid containing multiple caseous clots (Figure 1A), and was accompanied by fibrosis and hyperplasia of the adjacent glandular tissue. The mammary and internal iliac lymph nodes were enlarged, consistent with lymphoid hyperplasia. In case 2, the right cranial quarter was moderately enlarged, and the sinus contained multiple papillomatous and cystic intrasinusoidal proliferations, and the lumen was filled with serous fluid and caseous flakes (Figure 1B). The mammary lymph nodes were unremarkable. Both cows exhibited bilateral caudal accessory teats. The brachial plexuses of case 2 contained multiple, 2–6 mm in diameter, firm, beige nodules, histopathologically confirmed as benign peripheral nerve-sheath tumors.

### 2.3 Histopathology

**Figure 2:**
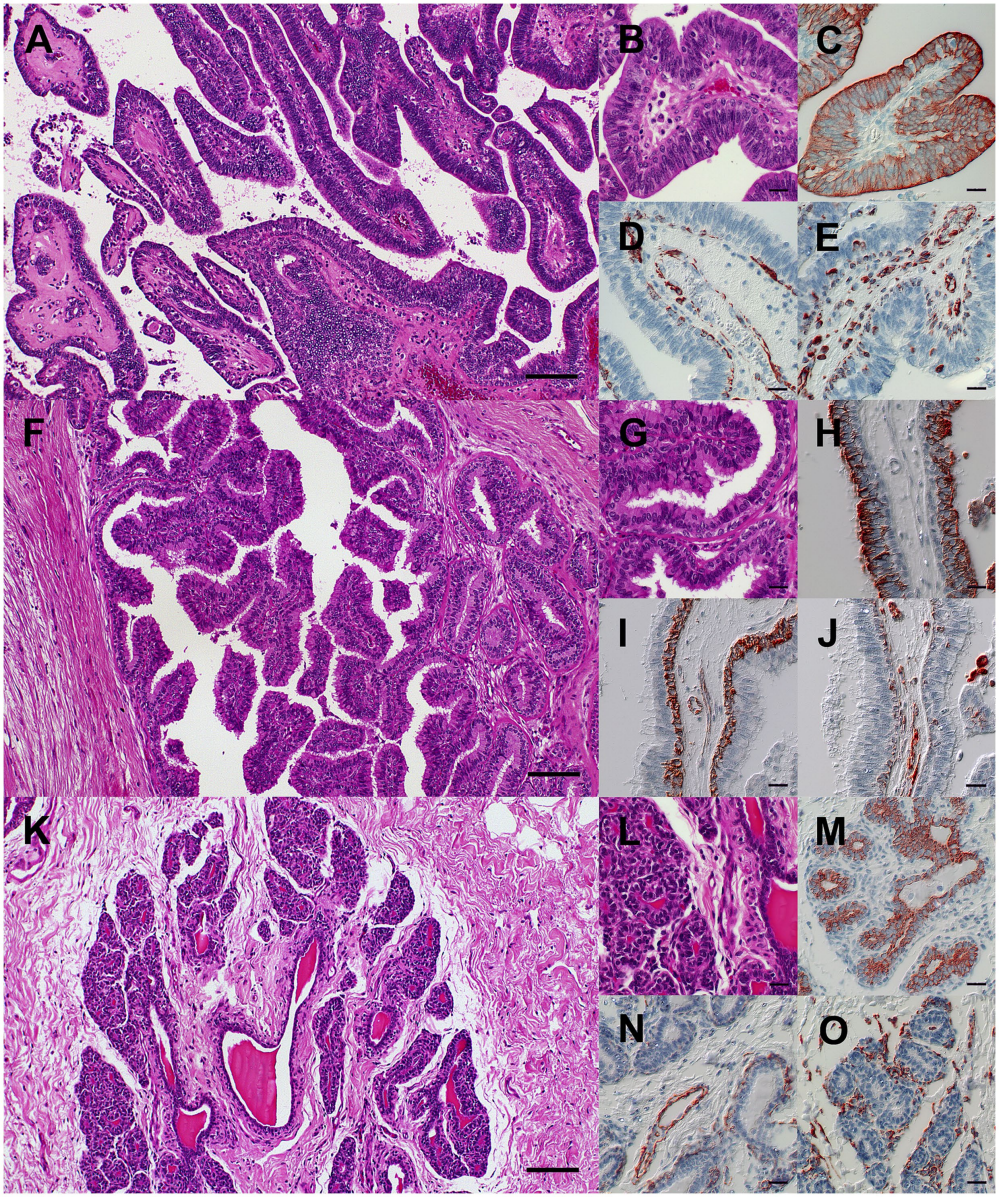
Histopathology and immunohistochemistry of two cases of multiple intraductal papillary adenomas of the bovine udder **(A–J)** compared with the normal non-lactating mammary gland **(K–O). (A–E)** Case 1, left cranial mammary quarter. **(A)** Papillary fronds with central fibrovascular, occasionally hyalinized stroma expanding into the lumen of the lactiferous sinus. **(B)** Papillae are lined by one to three layers of columnar epithelial cells and a basal layer of cuboidal myoepithelial cells. **(C)** Luminal epithelial cells display pan-cytokeratin immunoreactivity. **(D)** Basal myoepithelial cells and vascular smooth muscle cells exhibit smooth muscle actin immunoreactivity. **(E)** Basal myoepithelial and mesenchymal stromal cells, including fibroblasts and smooth muscle cells, show vimentin immunoreactivity. **(F–J)** Case 2, right cranial mammary quarter. **(F)** Papillary fronds extend into the lumen of a collecting duct adjacent to tubular structures surrounded by fibrovascular stroma (right side). **(G)** Papilla and tubules are lined by one to two layers of columnar epithelial cells and a basal layer of cuboidal myoepithelial cells. **(H)** Luminal epithelial cells displaying pan-cytokeratin immunoreactivity. **(I)** Basal myoepithelial cells and vascular smooth muscle cells exhibit smooth muscle actin immunoreactivity. **(J)** Basal myoepithelial and mesenchymal stromal cells, including fibroblasts and smooth muscle cells, show vimentin immunoreactivity. **(K–O)** Case 2, normal non-lactating mammary gland (unaffected left cranial quarter). **(K)** A central collecting duct is surrounded by poorly developed alveoli within an adipocyte-rich stroma, characteristic of the non-lactating gland. **(L)** Alveoli (left) and collecting ducts (right) are lined by a single layer of cuboidal epithelial cells and an indistinct and inconsistent layer of basal myoepithelial cells. **(M)** Alveolar and ductal epithelial cells display pan-cytokeratin immunoreactivity. **(N)** Smooth muscle actin–positive basal myoepithelial cells are more prominent around collecting ducts (right lower corner) than alveoli; the strongest staining is observed in vascular walls (center). **(O)** Basal myoepithelial and mesenchymal cells, including stromal fibroblasts and smooth muscle cells, show vimentin immunoreactivity. **(A,B,F,G,K,L)** Hematoxylin and eosin. **(C,H,M)** Pan-cytokeratin, **(D,I,N)** Smooth muscle actin, and **(E,J,O)** Vimentin immunohistochemistry using the avidin-biotin-peroxidase-complex method with 3-amino-9-ethyl-carbazol chromogen and hematoxylin counterstain. Bars: **A,F,K** = 100 μm; bars **B–E, G–J, L–O** = 20 μm.

Samples from the affected quarters were fixed in 4% neutral-buffered formaldehyde, embedded in paraffin, and sectioned at 2–4 μm. Sections were stained with hematoxylin and eosin (H&E) and periodic acid–Schiff (PAS). In case 1, the mass was poorly demarcated, non-encapsulated, and moderately cellular, expanding the ductal and sinusoidal spaces. Approximately 70% of the mass consisted of papillary fronds supported by fibrovascular stroma (Figure 2A), with the remainder forming tubules and acini embedded in collagen-rich connective tissue. The epithelial cells were arranged in a luminal layer of columnar cells and a basal layer of cuboidal cells, with distinct borders, moderately eosinophilic cytoplasm, as well as central to basal oval euchromatic nuclei with one to three nucleoli (Figure 2B). Mild to moderate anisocytosis and anisokaryosis were present. There were up to three mitoses per high-power field. Multifocal stromal hyalinization was observed. Luminal contents consisted of eosinophilic amorphous or foamy material mixed with viable and degenerate neutrophils and macrophages, and the stroma contained moderate multifocal to diffuse lymphohistioplasmacytic infiltrates, consistent with the clinical signs of chronic-active, catarrhal and suppurative mastitis. In case 2, sections showed a similar intraductal and intrasinusoidal papillary mass supported by a fibrovascular, and occasionally myxoid or hyalinized stroma (Figure 2F) with multifocal cystic dilation and fewer areas with tubuloacinar differentiation. The epithelium was arranged in a luminal layer of columnar cells and a basal layer of cuboidal cells (Figure 2G), with mild anisocytosis and anisokaryosis, and few foci of squamous metaplasia. There were up to three mitoses per high-power field. The stroma contained mild oligofocal lymphohistioplasmacytic infiltrates. In both cases, PAS reaction revealed continuous basement membranes separating the epithelial cells from the stroma. Furthermore, histopathological examination of the mammary lymph nodes, heart, lungs, spleen, liver, kidneys, and brain revealed no metastases. Comparative light microscopy of the normal left cranial mammary quarter of case 2 showed well-differentiated lobules of alveoli surrounding a central intralobular duct within an adipocyte-rich fibrovascular stroma (Figures 2K,L), serving as the reference for normal mammary architecture in this study.

### 2.4 Immunohistochemistry

Immunohistochemistry was performed using antibodies against cytokeratin, smooth-muscle actin, and vimentin with the avidin–biotin–peroxidase method. Antigen retrieval used citrate buffer (pH 6.0); 3-amino-9-ethylcarbazole served as chromogen and hematoxylin as counterstain.

Both cases displayed cytokeratin-immunoreactive luminal epithelial cells (Figures 2C,H), smooth muscle actin-immunoreactive basal myoepithelial cells and stromal vascular smooth muscles (Figures 2D,I), and vimentin-immunoreactive stromal fibroblasts and occasional myoepithelial cells (Figures 2E,J). No cytokeratin-positive cells were identified in lymph nodes or distant organs. Comparative normal mammary tissue from case 2 showed a typical ductal architecture with prominent smooth muscle actin-immunoreactive myoepithelial layers around ducts and reduced expression around alveoli (Figures 2M–O).

## 3 Discussion

The gross and microscopic findings were consistent with multiple benign epithelial neoplasms restricted to the ductal and sinusoidal system of the mammary gland in both cases. The absence of cellular and nuclear features of malignancy, necrosis, invasive growth and metastasis suggests a benign nature of the lesions. In agreement with the descriptions for canine and feline cases in the Davis–Thompson Foundation nomenclature (22) and the WHO/IARC classification (11, 23), both cases were diagnosed as multiple mammary intraductal papillary adenomas, characterized by intraluminal papillary projections with fibrovascular cores and an intact myoepithelial layer. These features clearly distinguished these neoplasms from the differential diagnosis of ductal adenoma, which lacks papillary architecture and instead displays bilayered cords or tubular structures within a dense stroma (22, 24). Immunohistochemistry corroborated the diagnosis. The bilayered epithelial–myoepithelial structure with cytokeratin and smooth muscle actin immunoreactivity confirmed benign glandular organization (11, 23, 25, 26). Vimentin expression in myoepithelial cells, occasionally with stromal hyalinization, likely reflected early stromal remodeling or myofibroblastic differentiation rather than malignant transformation (27–29). Intact PAS-positive basement membranes indicated non-invasive growth. These findings align with previous descriptions of benign mammary papillary adenomas in other species (11, 23, 25, 30). The purulent inflammation in case 1 was interpreted as the result of a suspected bacterial infection, which may have been predisposed by tumor-related obstruction of the milk ducts. Unfortunately, a microbiological examination of the milk could not be carried out due to the restrictions of the biosafety level 3** animal experiment. Notably, the concomitant occurrence of mammary neoplasms and mastitis has been reported previously in cattle (2), and while a causal role of chronic inflammation in tumor initiation remains unproven, an influence on neoplastic growth or biological behavior cannot be excluded.

Both cows were aged and nulliparous, and reproductive history may influence susceptibility. Parity and lactation promote terminal differentiation of mammary epithelium and are protective in human and small-animal oncology (16, 31, 32), although comparable data in cattle remain limited. Notably, mammary neoplasms are also rare in other large herbivores, such as mares and small ruminants, supporting a broader herbivore-associated resistance to mammary neoplasia (16–18). These parallels suggest shared endocrine, metabolic, or stem-cell regulatory mechanisms underlying natural resistance (9, 16). Despite their rarity, the bovine mammary gland closely resembles the human breast, and characterization of spontaneous neoplasms may provide meaningful insight into intrinsic neoplasia-resistance pathways and the broader mechanisms of mammary oncogenesis across species. Future studies should aim to establish and validate immunohistochemical assays to characterize the expression of key molecular markers of mammary neoplasms, such as estrogen and progesterone receptors and human epidermal growth factor receptor 2 in cattle, and to collect mammary gland specimens from neoplastic lesions as well as from nulliparous and multiparous control animals to assess the transcriptional activity of oncogenesis-related molecular pathways.

## 4 Conclusion

This report of two multiple mammary intraductal papillary adenomas in aged, nulliparous cows emphasizes the value of histopathology and immunophenotyping for accurate diagnosis and highlights the bovine mammary gland as a comparative model for mammary biology and oncogenesis across species.

## Data Availability

The original contributions presented in this case report are included in the article. Any further inquiries can be directed to the corresponding author.
